# Association between weather and utilisation of physical therapy in patients with osteoarthritis: a case-crossover study

**DOI:** 10.1186/s12891-022-05233-9

**Published:** 2022-03-19

**Authors:** Ruo-Yan Wu, Ren-Hao Pan, Chiung-Yi Wu, Chien-Lung Chan, Huan-Jui Yeh

**Affiliations:** 1 Department of Physical Medicine and Rehabilitation, Taoyuan General Hospital, Ministry of Health and Welfare, Taoyuan, Taiwan; 2 Department of Physical Therapy and Assistive Technology, National Yang Ming Chiao Tung University, Taipei, Taiwan; 3 Department of Information Management, Tunghai University, Taichung, Taiwan; 4 Community Medicine Research Center, National Yang Ming Chiao Tung University, Taipei, Taiwan; 5 La Vida Tec Co., Ltd., Taichung, Taiwan; 6 Department of Information Management, Yuan Ze University, Taoyuan, Taiwan; 7 Innovation Center for Big Data and Digital Convergence, Yuan Ze University, Taoyuan, Taiwan; 8 Institute of Public Health, National Yang Ming Chiao Tung University, Taipei, Taiwan

**Keywords:** Osteoarthritis, Physical therapy, Medical accessibility, Weather

## Abstract

**Background:**

During varied weather conditions, patients with osteoarthritis experience different severity of symptoms and signs. However, weather may also cause barriers or incentives for patients to seek medical services. These factors may result in changes in medical utilisation; however, no studies have investigated whether the probability of physical therapy utilisation among patients with osteoarthritis is associated with changes in meteorological factors.

**Method:**

By using a secondary data of NHID in Taiwan, we conducted a population-based, retrospective study with case-crossover design for patients initially diagnosed with osteoarthritis between 2000 and 2013. The meteorological factors of months with the lowest treatment rate were used as patients’ own control periods and compared with the parameters of months with high treatment frequency. The risk of exposure to different meteorological factors, including mean temperature, daily highest temperature, daily minimum temperature, diurnal temperature range, relative humidity, and barometric pressure, was estimated and represented by odds ratios (ORs) and 95% confidence intervals (CIs).

**Results:**

A total of 8,130 patients were recruited. Regardless of univariate or multivariable analysis, increased daily highest temperature enhanced the frequency of physical therapy (OR: 1.04; 95% CI: 1.02–1.05; *p* < 0.01; OR: 1.07; 95% CI: 1.04–1.10; *p* < 0.01). When the weather was hotter (> 23 °C), higher diurnal temperature range and humidity resulted in an increase in the utilisation of physical therapy. However, when the weather was colder (< 23 °C), reverse effects were observed.

**Conclusions:**

An increase in temperature increases the probability of physical therapy resource use. Therefore, temperature, along with other meteorological factors, may play a key role in the utilization of physical therapy among patients with osteoarthritis.

**Supplementary Information:**

The online version contains supplementary material available at 10.1186/s12891-022-05233-9.

## Introduction

With the increasing aging population worldwide, the number of patients with osteoarthritis (OA) is also increasing [1]. The prevalence rates of OA are approximately 13% to 33% in Eastern and Western countries [2, 3]. OA not only poses health problems for patients, but also creates huge social medical expenditures [3–5]. Physical therapy (PT) is an effective treatment in the initial stages of OA [6]. However, limited use of PT is associated with several factors such as being male, high OA severity, long duration of OA symptoms, poor general health, low income or low social economic status [7–10]. There is no consistent conclusion on the effect of age [7, 10]. These barriers may delay the treatment of patients with OA and lead to more complications in the future [11].

In addition to patient's individual factors, environmental factors may also affect the acceptance of PT, such as climate. First, climates may affect the severity of symptoms, such as increased pain, which may make patient desire to seek physical therapy [12]. Besides, the weather may affect the medical accessibility. For example, it is more difficult to reach an organization providing PT when it rains heavily.

About the influences on symptoms by weather, more than half of the OA patients mentioned that during weather changes, the intensity of joint stiffness or pain increased [13]. An increased barometric pressure may cause joint discomfort to become more evident [14, 15]. A possible reason is that the pressure pain threshold in OA patients is significantly lower than that in healthy people [16–19], and the altered barometric pressure causes changes in the synovial fluid of joints and decreases joint lubrication [20]. In contrast, a decrease in environmental temperature may increase the viscosity of synovial fluid and change the compliance of periarticular structures, thereby making joints stiffer and more sensitive to the pain of mechanical stresses produced by activities [21–24]. As bones, muscles, and tendons have different densities, humidity changes have also been found to affect the expansion and contraction of these tissues, which is associated with joint discomfort in OA patients [21, 25–27].

Weather not only affects the condition of OA symptoms, but also affects the ease or willingness of patients to seek medical treatment. Obvious events, such as heavy rains, typhoons, and heavy snow, will increase the difficulty for patients to get to the medical facility. Moreover, extreme hot or extreme cold weather may also decrease the willingness of patients to engage in outdoor activities. Therefore, the effect of weather on the utilisation of PT among OA patients is an issue that needs to be investigated.

This study combined regional weather data and consultation data to observe changes in the frequency of PT among OA patients during different weather conditions, to understand the association between the utilisation of PT resources and meteorological factors. Our hypothesis is that if the changes in treatment frequency are similar to the influence of climate on symptoms, the impact of climate on medical accessibility should be small. On the contrary, if the results are not in line with the expected changes in symptoms, it indicates that although patients potentially require more PT for symptoms control, they are more affected by the climate's influence which decreases their accessibility of PT or make them choose other treatments. Therefore, more effort should be made to reduce the medical barriers caused by weather.

## Methods

We conducted a retrospective study using data from a representative database of one million randomly selected patients (5% of the insured population) from the National Health Insurance Database (NHID) in 2005. This longitudinal data contains the health insurance data, including the consultation region, diagnostic codes, date of consultation, and patient information of selected patients from 1995 to 2013. The National Health Insurance (NHI) program in Taiwan is a mandatory general health insurance program in which the proportion of the insured population is greater than 99% of Taiwanese residents [11]. The database has disconnected public data which has been used greater than 1000 times in various health or medicine related studies. All methods were performed in accordance with the relevant guidelines and regulations. The study protocol complied with the provisions of the Declaration of Helsinki and was approved by the institutional review board of the hospital (IRB No: TYGH105040). Samples were selected from the database based on the International Classification of Diseases—9 (ICD-9) codes of patients and those with the ICD-9 code = 715.xx, were chosen as participants of the study. Patients who were initially diagnosed with OA between 2000–2013 and aged > 20 years were included in this study. Except for undergoing surgery, patients were not hospitalised for PT; therefore, hospitalised patients were excluded from this study.

**Fig. 1:**
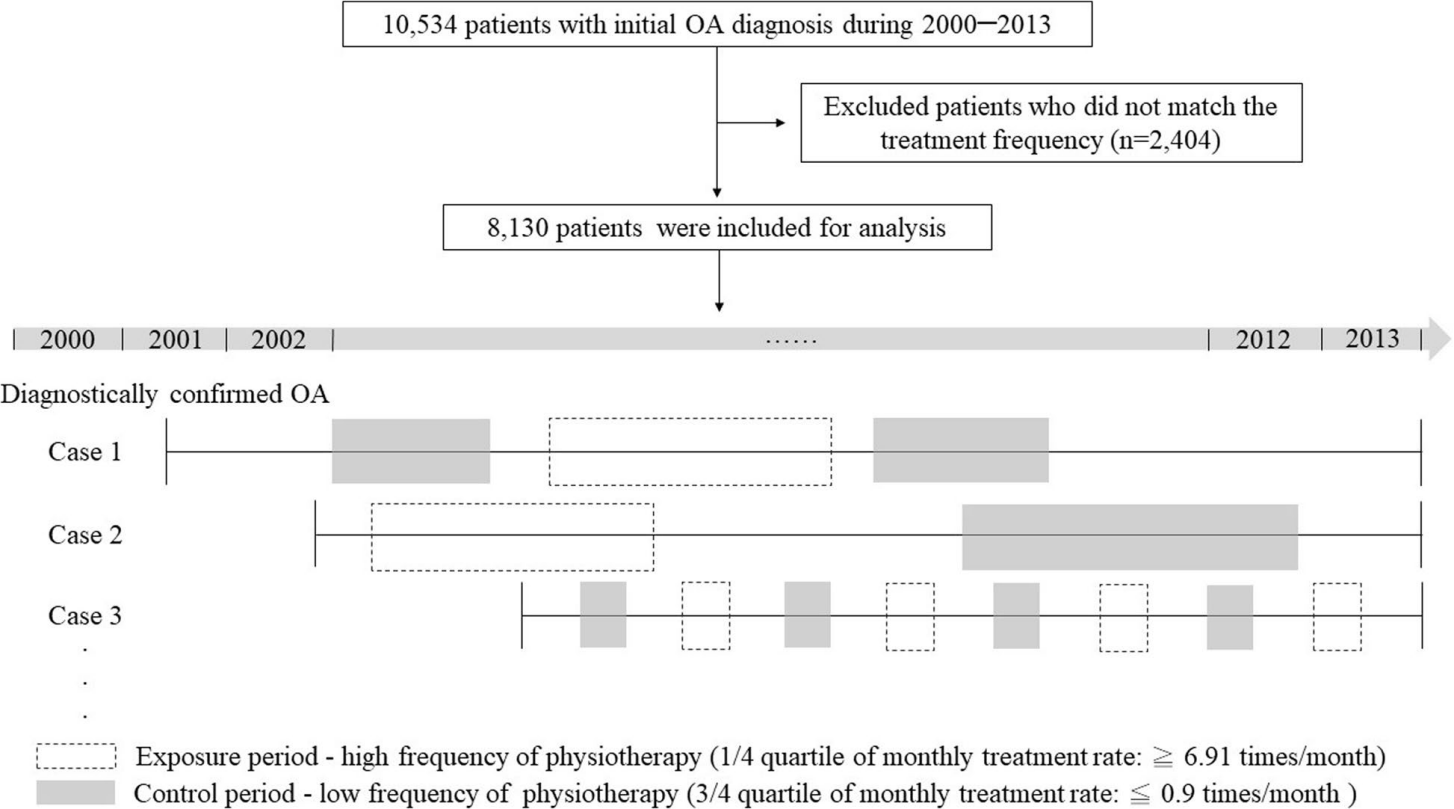
Flow chart of case and control months selection by case-crossover design

In addition to some factors that affect symptoms and accessibility for PT among OA patients (such as age, sex, weight, disease severity, income, location, and comorbidities), there are other factors that are difficult to measure, such as lifestyle habits, levels of daily activity, and psychological status, which may also affect their accessibility of PT resources. The confounding results of these individual factors are often the study limitations [28, 29]. Therefore, we used a case-crossover design by using patients as their own control group to correct for the unmeasurable confounders [30]. We segmented the average monthly treatment rate into quartiles and defined the top 25% as ‘high treatment frequency’ and lowest 25% as ‘low treatment frequency’. The month with the ‘lowest treatment frequency’ was used as the control period so that we could compare the meteorological factors between the high frequency and low frequency months of individual patients (Fig. 1).

The months when the patients sought consultation for OA were linked with the mean monthly values of various meteorological parameters. Meteorological data were collected from the Central Weather Bureau of Taiwan. Taiwan has 535 meteorological stations that record hourly measurements. Since PT for OA requires multiple trips to medical institutions and there are no large differences in treatment programs, most patients receive treatment at locations close to their home. Hence, we matched the nearest meteorological station (within 20 km) according to the location and date of consultation and calculated the following meteorological parameters: mean temperature (°C), daily highest temperature (°C), daily minimum temperature (°C), diurnal temperature range (°C), relative humidity (%), barometric pressure (hPa), and daily precipitation (mm).

The data analysis process was divided into three steps: 1) Analysing whether individual changes in each meteorological factor affected the treatment frequency. 2) Since weather is a combination of multiple meteorological factors simultaneously, we excluded factors with high collinearity (correlation coefficient > 0.5) and integrated the remaining meteorological factors to observe their effects on PT frequency. 3) Taiwan (our study region) is hotter than the countries where previously similar studies were conducted, and temperature was one of the often-mentioned factors that affected the symptoms [28]; thus, we further used the mean temperature as a cut-off point to divide all analysis data into two groups, and investigated the effects of individual and combined meteorological factors on treatment frequency under different temperatures. Furthermore, in order to ensure the reliability of our results, we also performed stratified analyses divided with tertile and quartile of daily mean temperature. The results of this part will be included in supplement files.

### Statistical Analysis

We used descriptive statistics to present the general information of patients and meteorological factors and used the correlation coefficient from Pearson’s correlation analysis to determine the possible collinearity between various meteorological factors. Subsequently, we used conditional logistic regression to analyse the correlation between meteorological factors and the treatment frequency of OA patients. This included univariate and multivariable analyses of the effects of every meteorological factor on the treatment frequency. We also used conditional logistic regression to analyse temperature stratification. The study results are presented as odds ratios (ORs) and 95% confidence intervals (CIs). OR represents the probability that the patient will undergo rehabilitation from a low frequency to a high frequency as each increased unit of climatic factors. For example, if the OR of daily mean temperature is 2, which means that as every 1 degree increase of daily mean temperature, the probability of receiving PT from low-frequency to high-frequency is double. Our study used Stata 12 statistical software for statistical analysis. Statistical significance was set at *p* < 0.05.

## Results

We extracted 10,534 patients from the NHIS database that met the diagnostic criteria with initial OA diagnosis. We used the quartiles of the treatment frequency as the cut-off points. When the number of PTs was more than 6.91 times per month, it was defined as higher treatment frequency and when it was lower than 0.90 times per month, it was defined as lower treatment frequency. After excluding patients who did not match the treatment frequency, 8,130 patients with 13,794 case months and 13,520 control months were finally collected from the NHID data for analysis. The average age of the patients was 59.78 (SD: 14.71), and 65% of the patients were women. Table 1 shows the meteorological data during the treatment period.

**Table 1:** Demographic characteristics of participants and meteorological factors

Characteristics (*n* = 8,130)	Mean ± SD or Number (%)			
**Age (years)**	59.78 ± 14.71			
**Gender: female (%)**	5,258 (65%)			
**Frequency of PT (times/month)**	5.07 ± 5.79	**Quartile**		
		25	50	75
		0.90	2.80	6.91
**Meteorological factors**	**Mean ± SD**			
**Mean temperature (°C)**	23.04 ± 4.77			
**Daily highest temperature (°C)**	31.35 ± 3.75			
**Daily minimum temperature (°C)**	20.62 ± 6.18			
**Diurnal temperature range (°C)**	2.97 ± 0.65			
**Relative humidity (%)**	76.74 ± 5.86			
**Barometric pressure (hPa)**	1,004.73 ± 15.78			
**Daily precipitation (mm)**	2.29 ± 2.19			

### Univariate analysis results

Regardless increase in the mean temperature, daily highest temperature, or daily minimum temperature, the treatment frequency of patients showed a significant increase (OR: 1.02, 95% CI: 1.01–1.04, *p* < 0.01; OR: 1.04, 95% CI: 1.02–1.05, *p* < 0.01; OR: 1.01, 95% CI: 1.01–1.02, *p* < 0.01). In addition, as barometric pressure increased, the treatment frequency decreased (OR: 0.99, 95% CI: 0.98–1.00, *p* = 0.03). There were no significant effects of diurnal temperature range, humidity, or daily precipitation on medical status (Table 2).

**Table 2:** Univariate analysis: meteorologic exposures and frequency of PT

	**Odds Ratio (95% CI)**	**SE**	**z**	***P***
Mean temperature (°C)	1.02 (1.01–1.04)	0.01	3.83	< 0.01*
Daily highest temperature (°C)	1.04 (1.02–1.05)	0.01	4.59	< 0.01*
Daily minimum temperature (°C)	1.01 (1.01–1.02)	0.01	3.09	< 0.01*
Diurnal temperature range (°C)	1.11 (1.00–1.23)	0.06	1.90	0.06
Relative humidity (%)	0.99 (0.98–1.01)	0.01	-0.76	0.45
Barometric pressure (hPa)	0.99 (0.98–1.00)	0.01	-2.13	0.03*
Daily precipitation (mm)	0.99 (0.96–1.01)	0.01	-0.94	0.35

^*^ *p* < 0.05

### Multivariable analysis results

Pearson’s correlation analysis showed that besides the correlation between mean temperature, daily highest temperature, and daily minimum temperature (correlation coefficient = 0.82 ~ 0.94), other meteorological factors showed low correlations with each other. To avoid issues with collinearity, we only included the daily highest temperature in the multivariable analysis (Table 3).

**Table 3:** Correlation coefficients between meteorological factors

	**Mean temperature**	**Daily highest temperature**	**Daily minimum temperature**	**Diurnal temperature range**	**Relative humidity**	**Barometric pressure**	**Daily precipitation**
Mean temperature	1	0.88**	0.94**	0.23**	-0.25**	-0.13**	0.45**
Daily highest temperature	0.88**	1	0.82**	0.32**	-0.15**	-0.27**	0.47**
Daily minimum temperature	0.94**	0.82**	1	0.25**	-0.23**	-0.24**	0.43**
Diurnal temperature range	0.23**	0.32**	0.25**	1	-0.37**	-0.07**	0.03**
Relative humidity	-0.25**	-0.15**	-0.23**	-0.37**	1	-0.28**	0.26**
Barometric pressure	-0.13**	-0.27**	-0.24**	-0.07**	-0.28**	1	-0.31**
Daily precipitation	0.45**	0.47**	0.43**	0.03**	0.26**	-0.31**	1

^**^ *p* < 0.01

When the daily highest temperature, diurnal temperature range, humidity, barometric pressure, and daily precipitation were analysed together, it was found that when the daily highest temperature increased, the frequency of PT also increased (OR: 1.07, 95% CI: 1.04–1.10, *p* < 0.01). In addition, when the daily precipitation increased, the frequency of PT decreased (OR: 0.95, 95% CI: 0.92–0.98, *p* < 0.01). The diurnal temperature range, relative humidity, and barometric pressure did not show any significant effects (Table 4).

**Table 4:** Multivariable analysis: meteorologic exposures and frequency of PT

	**Odds Ratio (95% CI)**	**SE**	**Z**	***P***
Daily highest temperature (∘C)	1.07 (1.04–1.10)	0.01	4.89	< 0.01*
Diurnal temperature range (∘C)	1.01 (0.89–1.15)	0.07	0.13	0.89
Relative humidity (%)	1.01 (1.00–1.02)	0.01	1.28	0.20
Barometric pressure (hPa)	1.01 (1.00–1.03)	0.01	1.47	0.14
Daily precipitation (mm)	0.95 (0.92–0.98)	0.02	-2.99	< 0.01*

^*^ *p* < 0.05

When the daily highest temperature, diurnal temperature range, humidity, barometric pressure, and daily precipitation were analysed together, it was found that when the daily highest temperature increased, the frequency of PT also increased (OR: 1.07, 95% CI: 1.04–1.10, *p* < 0.01). In addition, when the daily precipitation increased, the frequency of PT decreased (OR: 0.95, 95% CI: 0.92–0.98, *p* < 0.01). The diurnal temperature range, relative humidity, and barometric pressure did not show any significant effects (Table 4).

### Temperature stratification results

Under different temperature conditions, diurnal temperature range and relative humidity affected the treatment frequency. The mean temperature value was 23 °C, which was used as a cut-off point for hot and cold weather. Univariate analysis revealed that during hot weather, increased diurnal temperature range increased the treatment frequency (OR: 1.25, 95% CI: 1.03–1.52, *p* = 0.03). Multivariable analysis showed that the higher the diurnal temperature range and humidity, the higher the treatment frequency (OR: 1.48, 95% CI: 1.15–1.90, *p* < 0.01; OR: 1.05, 95% CI: 1.02–1.08, *p* < 0.01). Univariate analysis found that during cold weather, increased humidity resulted in decreased treatment frequency (OR: 0.98, 95% CI: 0.96–1.00, *p* = 0.03). When other meteorological factors were combined, increases in the diurnal temperature range and humidity resulted in a decrease in treatment frequency (OR: 0.66, 95% CI: 0.54–0.82, *p* < 0.01; OR: 0.97, 95% CI: 0.94–0.99, *p* < 0.01) (Table 5).

**Table 5:** Univariate and Multivariable analysis (23 °C used as a cut-off point): meteorologic exposures and frequency of PT

Temperature stratification		Univariate (non-adjust) analysis		Multivariable (adjust) analysis	
		**Odds Ratio (95% CI)**	***P* value**	**Odds Ratio (95% CI)**	***P* value**
Mean temperature > 23 °C	Daily highest temperature	1.02 (0.97–1.07)	0.43	1.01 (0.94–1.08)	0.75
	Diurnal temperature range	1.25 (1.03–1.52)	0.03*	1.48 (1.15–1.90)	< 0.01*
	Relative humidity	1.02 (1.00–1.04)	0.09	1.05 (1.02–1.08)	< 0.01*
	Barometric pressure	1.00 (0.98–1.02)	0.99	1.01 (0.99–1.04)	0.31
	Daily precipitation	0.99 (0.95–1.02)	0.40	0.96 (0.92–1.00)	0.05
Mean temperature < 23 °C	Daily highest temperature	1.05 (1.02–1.08)	< 0.01*	1.09 (1.04–1.13)	< 0.01*
	Diurnal temperature range	0.88 (0.74–1.04)	0.14	0.66 (0.54–0.82)	< 0.01*
	Relative humidity	0.98 (0.96–1.00)	0.03*	0.97 (0.94–0.99)	< 0.01*
	Barometric pressure	1.00 (0.98–1.02)	0.94	1.01 (0.98–1.04)	0.36
	Daily precipitation	0.96 (0.89–1.03)	0.26	0.93 (0.85–1.02)	0.11

^*^ *p* < 0.05

Supplementary information: Stratified multivariable analysis (dividing the regions into 3 groups and 4 groups by tertile and quartile of mean temperature as a cut-off point): meteorologic exposures and frequency of PT. The results were showed in Supplement 1 & Supplement 2.

## Discussion

This study investigated the influence of weather changes in tropical and subtropical regions on PT resource utilisation among OA patients. We found that higher temperatures increased PT utilisation among OA patients. Barometric pressure, and daily precipitation had inconsistent effect on PT utilisation under different analyses, and its impact was smaller than the influence of temperature. Additionally, the effects of other meteorological factors altered with the mean temperature changes. In hotter weather (> 23 °C), higher diurnal temperature range and humidity resulted in increased PT use. However, in colder weather (< 23 °C), reverse effects were observed.

Previous studies have found that cold weather could result in worsened symptoms [21, 22, 29, 31]. In addition to its effect on the musculoskeletal system, temperature drop increased the pain perception of the central nervous system [32]. With regards to the effects of barometric pressure on OA, Peultier et al. compiled relevant articles and found seven articles that revealed that there was a significant correlation between barometric pressure and symptoms. However, the results of these studies were not consistent in that some found that increased barometric pressure resulted in greater symptom severity, while other studies found that low barometric pressure aggravated pain [28]. Another study revealed that an increased relative humidity increased the Western Ontario McMaster Universities Osteoarthritis Index (WOMAC) pain score among OA patients [29]. Moreover, a previous study showed that there were significant associations between joint pain and daily average humidity, and this effect increased during cold conditions [25].

As mentioned above, OA patients should experience worsening symptoms when the weather is colder or when humidity is higher. OA patients require more PT at these times. On the contrary, in our research, we found that patients reduced their use of PT during cold weather as well as high humidity conditions. These findings indicate that the frequency of PT among OA patients may be most affected by medical accessibility, rather than temporary changes in symptoms.

How does weather affect medical accessibility? Previous studies have focused on two aspects. First, extreme weather or catastrophic climate increases the difficulty of transportation and affects the convenience of outdoor activities to reach medical institutions [33]. Bad weather, such as extreme heat, rainy days and typhoons, may decrease the patients’ desire to go out and seek regular outpatient treatment [34]. Second, under adverse weather conditions, the rapidly increased number of patients availing medical treatment may lead to shortage of resource and further hinder other people to access healthcare services [35]. However, these theories seem to be insufficient to explain why changes in temperature and humidity affect the use of PT in Taiwan.

Rain may affect the convenience of transportation or decrease the willingness to leave home for medical treatment. But these findings were not robust in our study. The possible reasons are as follows: 1. Daily precipitation does not represent how long it rained and when it rained. In Taiwan, people can choose anytime to receive PT during operating hours of medical hospitals or clinics, even without advance appointment. Due to this reason, if it doesn’t rain for a whole day and night, patients can still go out for treatment when the rain stops. 2. Hospital or clinics for rehabilitation are extremely dense in Taiwan. The inconvenience caused by rain may be reduced by the short distance from home to hospital.

In Taiwan, patients usually receive PT anytime they want without an appointment. Therefore, relatively uncomfortable weather may cause patients to choose another day for treatment. In addition, since Taiwan is located at the border of subtropical and tropical regions, most air-conditioning systems in hospitals provide cooling rather than heating functions. In scorching weather, the air-conditioned hospital environment provides patients with a greater incentive to receive treatment. In contrast, cold and clammy weather makes patients less willing to go out, and their discomfort is not relieved by arriving at the medical facility. Therefore, even if patients experience more physical discomfort, they do not utilise PT. Another reason why patients avail their PT treatment on days with higher daily highest temperature during cold weather conditions is that the weather is relatively more comfortable for patients to go outside.

Although pain and joint stiffness decrease the walking ability and endurance, which may reduce accessibility of medical services, worsening symptoms should be a stronger driving force for patients to receive treatment. The PT utilisation, contrary to clinical symptoms, indicates that the greatest influence on the utilisation of PT among OA patients is not based on the severity of symptoms, rather it is external weather conditions. Future studies may explore whether PT utilisation in regions with relatively adequate medical treatment is restricted by medical accessibility or subject to overuse.

### Study Limitations

This is a nationwide study with the advantage that health insurance coverage is as high as 99%. Therefore, sufficient patient consultation data were obtained. In addition, this study used a case-crossover design to exclude measurable and unmeasurable confounding variables. However, although case-crossover study design can help us eliminate the interference caused by individual differences, the new changes that individuals produce during the overall study period may still interfere the results. This study has the following limitations: 1) The individual exposure may not be completely consistent with the environment of the meteorological station. Moreover, we were also unable to confirm whether the patients spent the majority of their time in air-conditioned environments. However, the coverage of meteorological stations in Taiwan is very extensive, and the reliability of the data should be higher than those observed in previous studies. 2) Because weather may change a lot during a month, our results can only explain the tendency of patients to receive physical therapy in a wider range of climatic conditions, rather than the impact from immediate weather changes. We need further studies to investigate the influence of weather fluctuations on the accessibility of PT. 3) The effects of temperature on accessibility of PT may tend to be non-linear, and our analysis model may not be completely suitable. However, we used stratified analysis to explore the effects of various meteorological factors under different temperature ranges. And it should be able to express the influence of various meteorological factors under different conditions. 4) The climate of Taiwan tends to be hotter than most other countries, hence, the results may not be extrapolated to other non-tropical regions. We recommend that future studies investigate the effects of meteorological factors in low temperature conditions, which may provide a better understanding of the effects of various meteorological factors on PT utilisation. 5) The utilisation of medical resources varies greatly among different medical insurance systems. Medical resources are relatively cheaper and easier to obtain for patients in Taiwan. Therefore, our research results should only be analogous to countries with relatively sufficient rehabilitation medical resources.

## Conclusion

In tropical and subtropical regions, increases in temperature may be associated with increased PT utilisation among OA patients. During hotter weather, increases in diurnal temperature range and humidity are associated with increased PT use. In colder weather, increases in diurnal temperature range and humidity are associated with less PT use. This result is contrary to our perception of the influence of weather on OA symptoms, which may indicate that the impact of climate on medical accessibility is greater than the influence of the symptoms. More studies are required to prove the effects of various meteorological factors on PT utilisation under different temperature conditions.

## Supplementary Information

**Additional file 1.**

**Additional file 2.**

## Data Availability

The data that supports the findings of this study is accessible from the Health and Welfare Data Science Center of Taiwan. However, because restrictions apply to the accessibility of said data, they are not publicly available. Nevertheless, the data is available from the authors upon reasonable request and with permission of the Health and Welfare Data Science Center of Taiwan.

## Abbreviations

OA: Osteoarthritis
ICD: International Classifications of Diseases
OR: Odds ratio
CI: Confidence interval
NHID: National Health Insurance Database
NHI: National Health Insurance
WOMAC: Western Ontario McMaster Universities Osteoarthritis Index

## References

[1] Cross M, Smith E, Hoy D, Nolte S, Ackerman I, Fransen M, Bridgett L, Williams S, Guillemin F, Hill CL (2014). The global burden of hip and knee osteoarthritis: estimates from the global burden of disease 2010 study. Ann Rheum Dis.

[2] White AG, Birnbaum HG, Janagap C, Buteau S, Schein J (2008). Direct and indirect costs of pain therapy for osteoarthritis in an insured population in the United States. J Occup Environ Med.

[3] Le TK, Montejano LB, Cao Z, Zhao Y, Ang D (2012). Health care costs in US patients with and without a diagnosis of osteoarthritis. J Pain Res.

[4] Chen A, Gupte C, Akhtar K, Smith P, Cobb J (2012). The Global Economic Cost of Osteoarthritis How the UK Compares. Arthritis.

[5] Kotlarz H, Gunnarsson CL, Fang H, Rizzo JA (2009). Insurer and out-of-pocket costs of osteoarthritis in the US: evidence from national survey data. Arthritis Rheum.

[6] Hochberg MC, Altman RD, April KT, Benkhalti M, Guyatt G, McGowan J, Towheed T, Welch V, Wells G, Tugwell P (2012). American College of Rheumatology 2012 recommendations for the use of nonpharmacologic and pharmacologic therapies in osteoarthritis of the hand, hip, and knee. Arthritis Care Res (Hoboken).

[7] Yeh HJ, Chou YJ, Yang NP, Huang N (2015). Receipt of physical therapy among osteoarthritis patients and its influencing factors. Arch Phys Med Rehabil.

[8] Iversen MD, Schwartz TA, von Heideken J, Callahan LF, Golightly YM, Goode A, Hill C, Huffman K, Pathak A, Cooke J (2018). Sociodemographic and Clinical Correlates of Physical Therapy Utilization in Adults With Symptomatic Knee Osteoarthritis. Phys Ther.

[9] Jacobs H, Seeber GH, Allers K, Hoffmann F (2021). Utilisation of outpatient physiotherapy in patients following total knee arthroplasty - a systematic review. BMC Musculoskelet Disord.

[10] Sussmann KE JH, Hoffmann F (2021). Physical Therapy Use and Associated Factors in Adults with and without Osteoarthritis-An Analysis of the Population-Based German Health Update Study. Healthcare (Basel).

[11] Yeh HJ, Chou YJ, Yang NP, Cheng CC, Huang N (2016). Association Between Physical Therapy and Risk of Coronary Artery Disease and Dyslipidemia Among Osteoarthritis Patients: A Nationwide Database Study. Arch Phys Med Rehabil.

[12] Dibonaventura MD, Gupta S, McDonald M, Sadosky A, Pettitt D, Silverman S (2012). Impact of self-rated osteoarthritis severity in an employed population: cross-sectional analysis of data from the national health and wellness survey. Health Qual Life Outcomes.

[13] Sibley JT (1985). Weather and arthritis symptoms. J Rheumatol.

[14] Hunter DJ, Riordan EA (2014). The impact of arthritis on pain and quality of life: an Australian survey. Int J Rheum Dis.

[15] Moss P, Knight E, Wright A (2016). Subjects with Knee Osteoarthritis Exhibit Widespread Hyperalgesia to Pressure and Cold. PLoS One.

[16] Suokas AK, Walsh DA, McWilliams DF, Condon L, Moreton B, Wylde V, Arendt-Nielsen L, Zhang W (2012). Quantitative sensory testing in painful osteoarthritis: a systematic review and meta-analysis. Osteoarthritis Cartilage.

[17] Arendt-Nielsen L, Nie H, Laursen MB, Laursen BS, Madeleine P, Simonsen OH, Graven-Nielsen T (2010). Sensitization in patients with painful knee osteoarthritis. Pain.

[18] Imamura M, Imamura ST, Kaziyama HH, Targino RA, Hsing WT, de Souza LP, Cutait MM, Fregni F, Camanho GL (2008). Impact of nervous system hyperalgesia on pain, disability, and quality of life in patients with knee osteoarthritis: a controlled analysis. Arthritis Rheum.

[19] Wylde V, Palmer S, Learmonth ID, Dieppe P (2012). Somatosensory abnormalities in knee OA. Rheumatology (Oxford).

[20] Brennan SA, Harney T, Queally JM, O'Connor McGoona J, Gormley IC, Shannon FJ (2012). Influence of weather variables on pain severity in end-stage osteoarthritis. Int Orthop.

[21] Jamison RN, Anderson KO, Slater MA (1995). Weather changes and pain: perceived influence of local climate on pain complaint in chronic pain patients. Pain.

[22] Golde B (1992). New clues into the etiology of osteoporosis: the effects of prostaglandins (E2 and F2 alpha) on bone. Med Hypotheses.

[23] Robbins SM, Jones GR, Birmingham TB, Maly MR (2013). Quantity and quality of physical activity are influenced by outdoor temperature in people with knee osteoarthritis. Physiother Can.

[24] Feinglass J, Lee J, Semanik P, Song J, Dunlop D, Chang R (2011). The effects of daily weather on accelerometer-measured physical activity. J Phys Act Health.

[25] Timmermans EJ, Schaap LA, Herbolsheimer F, Dennison EM, Maggi S, Pedersen NL, Castell MV, Denkinger MD, Edwards MH, Limongi F (2015). The Influence of Weather Conditions on Joint Pain in Older People with Osteoarthritis: Results from the European Project on OSteoArthritis. J Rheumatol.

[26] Aikman H (1997). The association between arthritis and the weather. Int J Biometeorol.

[27] Strusberg I, Mendelberg RC, Serra HA, Strusberg AM (2002). Influence of weather conditions on rheumatic pain. J Rheumatol.

[28] Peultier L, Lion A, Chary-Valckenaere I, Loeuille D, Zhang Z, Rat AC, Gueguen R, Paysant J, Perrin PP (2017). Influence of meteorological elements on balance control and pain in patients with symptomatic knee osteoarthritis. Int J Biometeorol.

[29] Dorleijn DM, Luijsterburg PA, Burdorf A, Rozendaal RM, Verhaar JA, Bos PK, Bierma-Zeinstra SM (2014). Associations between weather conditions and clinical symptoms in patients with hip osteoarthritis: a 2-year cohort study. Pain.

[30] Maclure M, Mittleman MA (2000). Should we use a case-crossover design?. Annu Rev Public Health.

[31] McAlindon T, Formica M, Schmid CH, Fletcher J (2007). Changes in barometric pressure and ambient temperature influence osteoarthritis pain. Am J Med.

[32] Bongers J, Vandenneucker H (2020). The influence of weather conditions on osteoarthritis and joint pain after prosthetic surgery. Acta Orthop Belg.

[33] Blanford JI, Kumar S, Luo W, MacEachren AM (2012). It's a long, long walk: accessibility to hospitals, maternity and integrated health centers in Niger. Int J Health Geogr.

[34] Samano D, Saha S, Kot TC, Potter JE, Duthely LM (2021). Impact of Extreme Weather on Healthcare Utilization by People with HIV in Metropolitan Miami. Int J Environ Res Public Health.

[35] Bishop-Williams KE, Berrang-Ford L, Sargeant JM, Pearl DL, Lwasa S, Namanya DB, Edge VL, Cunsolo A, IR Team, Bwindi Community Hospital (2018). Understanding Weather and Hospital Admissions Patterns to Inform Climate Change Adaptation Strategies in the Healthcare Sector in Uganda. Int J Environ Res Public Health.

