# Supplementary material for: Association between weather and utilisation of physical therapy in patients with osteoarthritis: a case-crossover study
Source: BMC Musculoskelet Disord. 2022 Mar 19;23:269. doi: 10.1186/s12891-022-05233-9 (PMC8933890; doi:10.1186/s12891-022-05233-9)
Supplement: Supplementary file 1 — Additional file 1. [file 12891_2022_5233_MOESM1_ESM.docx]

Supplement 1: Multivariate analysis (tertile of mean temperature as a cut-off point): meteorologic exposures and frequency of physiotherapy

| Temperature stratification | | Odds Ratio (95% CI) | SE | z | P |
| --- | --- | --- | --- | --- | --- |
| Mean temperature >26.14∘C | Daily highest temperature | 1.09 (0.96-1.23) | 0.07 | 1.26 | 0.21 |
|  | Diurnal temperature range | 1.69 (1.06-2.69) | 0.40 | 2.20 | <0.03* |
|  | Relative humidity | 1.06 (1.02-1.11) | 0.02 | 2.98 | <0.01* |
|  | Barometric pressure | 1.05 (0.99-1.11) | 0.03 | 1.51 | 0.13 |
|  | Precipitation (mm) | 0.96 (0.90-1.03) | 0.03 | -1.19 | 0.23 |
| 20.52∘C < Mean temperature  ≦26.14∘C | Daily highest temperature | 0.99 (0.90-1.08) | 0.04 | -0.31 | 0.76 |
|  | Diurnal temperature range | 1.00 (0.74-1.37) | 0.16 | 0.06 | 0.95 |
|  | Relative humidity | 1.01 (0.97-1.05) | 0.02 | 0.47 | 0.64 |
|  | Barometric pressure | 1.00 (0.97-1.02) | 0.01 | -0.28 | 0.78 |
|  | Precipitation (mm) | 0.95 (0.89-1.02) | 0.03 | -1.30 | 0.19 |
| Mean temperature ≦20.52 ∘C | Daily highest temperature | 1.09 (1.03-1.16) | 0.03 | 3.03 | <0.01* |
|  | Diurnal temperature range | 0.68 (0.51-0.90) | 0.10 | -2.65 | <0.01* |
|  | Relative humidity | 0.95 (0.92-0.98) | 0.01 | -3.49 | <0.01* |
|  | Barometric pressure | 1.02 (0.98-1.06) | 0.02 | 0.93 | 0.35 |
|  | Precipitation (mm) | 0.97 (0.85-1.10) | 0.06 | -0.47 | 0.64 |

* p<0.05
