# Supplementary material for: Association between weather and utilisation of physical therapy in patients with osteoarthritis: a case-crossover study
Source: BMC Musculoskelet Disord. 2022 Mar 19;23:269. doi: 10.1186/s12891-022-05233-9 (PMC8933890; doi:10.1186/s12891-022-05233-9)
Supplement: Supplementary file 2 — Additional file 2. [file 12891_2022_5233_MOESM2_ESM.docx]

Supplement 2: Multivariate analysis (quartile of mean temperature as a cut-off point): meteorologic exposures and frequency of physiotherapy

| Temperature stratification | | Odds Ratio (95% CI) | SE | z | P |
| --- | --- | --- | --- | --- | --- |
| Mean temperature > 27.54∘C | Daily highest temperature | 1.10 (0.92-1.31) | 0.10 | 1.02 | 0.31 |
|  | Diurnal temperature range | 1.27 (0.65-2.48) | 0.43 | 0.70 | 0.48 |
|  | Relative humidity | 1.05 (0.99-1.11) | 0.03 | 1.71 | 0.09 |
|  | Barometric pressure | 1.02 (0.93-1.12) | 0.05 | 0.49 | 0.62 |
|  | Precipitation (mm) | 0.94 (0.87-1.03) | 0.04 | -1.37 | 0.17 |
| 23.52∘C < Mean temperature  ≦27.54∘C | Daily highest temperature | 0.99 (0.86-1.14) | 0.07 | -0.13 | 0.90 |
|  | Diurnal temperature range | 1.16 (0.75-1.81) | 0.26 | 0.68 | 0.50 |
|  | Relative humidity | 1.05 (1.00-1.09) | 0.02 | 2.13 | 0.03* |
|  | Barometric pressure | 1.02 (0.98-1.06) | 0.02 | 0.89 | 0.37 |
|  | Precipitation (mm) | 0.92 (0.85-0.99) | 0.04 | -2.13 | 0.03* |
| 18.96∘C < Mean temperature  ≦23.52∘C | Daily highest temperature | 1.00 (0.91-1.10) | 0.05 | 0.03 | 0.97 |
|  | Diurnal temperature range | 0.92 (0.65-1.29) | 0.16 | -0.51 | 0.61 |
|  | Relative humidity | 0.98 (0.94-1.02) | 0.02 | -0.94 | 0.35 |
|  | Barometric pressure | 1.00 (0.96-1.05) | 0.02 | 0.17 | 0.87 |
|  | Precipitation (mm) | 0.96 (0.86-1.07) | 0.06 | -0.72 | 0.47 |
| Mean temperature ≦18.96 ∘C | Daily highest temperature | 1.10 (1.03-1.18) | 0.04 | 2.70 | <0.01* |
|  | Diurnal temperature range | 0.57 (0.40-0.82) | 0.10 | -3.04 | <0.01* |
|  | Relative humidity | 0.94 (0.90-0.97) | 0.02 | -3.56 | <0.01* |
|  | Barometric pressure | 0.99 (0.96-1.03) | 0.02 | -0.28 | 0.78 |
|  | Precipitation (mm) | 1.02 (0.87-1.20) | 0.08 | 0.26 | 0.80 |

* p<0.05
